# In This Issue

**Published:** 2000

## EPIDEMIOLOGIC ANALYSIS OF ALCOHOL AND TOBACCO USE

Recent epidemiologic studies using data from nationwide surveys have investigated the prevalence of co-occurring alcohol and tobacco use and dependence, write Drs. James C. Anthony and Fernando Echeagaray-Wagner. Analyses based on the National Household Survey on Drug Abuse found that alcohol and/or tobacco use was least common among young adolescents, peaked among young adults, and declined thereafter. Moreover, a majority of smokers reported that they consumed alcohol, whereas a smaller proportion of alcohol users reported smoking. Data from the National Comorbidity Survey showed that the prevalence of alcohol dependence declined with increasing age. Conversely, the prevalence of tobacco dependence increased among young adult smokers and remained high until declining again among the oldest segment of the study population. The prevalence rates of concurrent alcohol and tobacco dependence were similar to the rates for tobacco dependence; that is, combined alcohol and tobacco dependence appeared to be less prevalent among 15- to 18-year-old users than among both older adolescent and young adult users. (pp. 201–208)

## SMOKING AND THE GENETIC CONTRIBUTION TO ALCOHOL DEPENDENCE RISK

Genetics influences a person’s risk of becoming dependent on nicotine as well as his or her risk of becoming dependent on alcohol. Because substantially higher rates of smoking are observed in alcoholics than in control groups, uncovering the mechanisms underlying this association may have important implications for both treatment and prevention. In this article, Drs. Pamela A. F. Madden, Kathleen K. Bucholz, Nicholas G. Martin, and Andrew C. Heath review data from studies conducted in twins to show that a positive genetic correlation exists between smoking and the risk of alcohol dependence. This correlation holds true even when researchers consider both sociodemographic and personality variables as well as the histories of other psychopathologies. (pp. 209–214)

## BEHAVIORAL MECHANISMS UNDERLYING THE LINK BETWEEN SMOKING AND DRINKING

What prompts someone to begin using alcohol and tobacco? According to Dr. Hilary J. Little, similar factors may contribute to the initiation of alcohol and tobacco use. Both drugs have been suggested to have anxiety-reducing and antidepressant effects, and both drugs may induce euphoria and other rewarding responses. A person’s level of impulsivity and sensation seeking also may influence his or her propensity to begin using alcohol and tobacco. This article reviews the mechanisms that may underlie alcohol and tobacco dependence. Specific mechanisms, such as those contributing to the development of tolerance and sensitization to the drugs’ effects, may overlap to some extent for alcohol and nicotine. (pp. 215–224)

## SOCIOCULTURAL INFLUENCES ON SMOKING AND DRINKING

Research indicates that sociocultural factors influence the initiation and continued use of alcohol and tobacco among adolescents and adults. In adolescents, for example, those sociocultural risk factors may include family and peer influences, demographic factors, and economic and availability factors. Few studies have examined how sociocultural factors affect drinking among smokers and smoking among drinkers. However, the limited evidence available suggests that such factors do have an impact and that the strength of the association between alcohol and tobacco use varies according to the amount of alcohol consumed. Drs. Janet Kay Bobo and Corinne Husten review research on the sociocultural factors that influence whether adolescents initiate alcohol and tobacco use and then discuss similar factors that may sustain alcohol and tobacco use among adults. The authors also show how public health interventions that focus on concurrent tobacco and alcohol use may help reduce the morbidity and mortality associated with these substances. (pp. 225–232)

## CO-OCCURRING RISK FACTORS FOR ALCOHOL DEPENDENCE AND HABITUAL SMOKING

The Collaborative Study on the Genetics of Alcoholism (COGA), a multicenter study analyzing the genetic factors contributing to alcohol dependence, also has given researchers the opportunity to investigate the genetic factors that may determine risk for habitual smoking. Drs. Laura Jean Bierut, Marc A. Schuckit, Victor Hesselbrock, and Theodore Reich report that the COGA study has provided evidence that risk for alcoholism and habitual smoking may be inherited. People with alcoholic parents reported substantially higher rates of both alcohol dependence and habitual smoking than did those with parents who were not alcohol dependent. COGA researchers are using advanced techniques (i.e., candidate gene studies and genomic screening) to identify the factors that may be behind this genetic link. Such investigations have located a number of regions on the human chromosomes that may contain candidate genes contributing to the risk for alcohol dependence, some of which also may contribute to the risk of habitual smoking. (pp. 233–241)

## THE EFFECTS OF TOBACCO USE DURING AND AFTER PREGNANCY ON EXPOSED CHILDREN

It is well documented that the use of tobacco and alcohol during pregnancy is associated with a number of adverse effects on the growth, cognitive development, and behavior of the exposed child. Despite this fact, many women continue to smoke and drink during pregnancy. Drs. Marie D. Cornelius and Nancy L. Day describe the prevalence of alcohol and tobacco use during pregnancy and review findings on the effects of maternal smoking during pregnancy as well as the effects of pre-natal and postnatal exposure to “secondhand” smoke. Understanding the effects of prenatal tobacco exposure allows researchers to identify those characteristics that are uniquely related to tobacco and those that are affected by alcohol exposure. This research, along with research on the effects of alcohol use during pregnancy, has implications for preventing various types of substance use during pregnancy and for treating children affected by prenatal substance use. (pp. 242–249)

## PREVENTING ALCOHOL AND TOBACCO USE THROUGH LIFE SKILLS TRAINING

Surveys indicate that rates of alcohol and tobacco use increase among high school students as they age. Prevention programs that target youth either before or during junior high school may help prevent such substance use during high school. Dr. Gilbert J. Botvin and Ms. Lori Wolfgang Kantor describe one school-based program—Life Skills Training (LST)—designed to prevent substance abuse among youth by influencing risk factors associated with the early stages of alcohol, tobacco, and other drug use. The authors describe the rationale behind the LST approach, discuss the program’s components and their implementation, and review research evaluating the effectiveness of the program. Early LST research focused on tobacco use and involved predominantly white middle-class populations. However, more recent research has examined the use of the LST approach in preventing alcohol, marijuana, and other illicit drug use. Recent research also has evaluated the LST approach among minority populations and has assessed the long-term durability of LST as a tool for the prevention of substance abuse in adolescents. (pp. 250–257)

